# Editorial: Epilepsy in older adults: From physiopathology to improvements in diagnosis and management

**DOI:** 10.3389/fneur.2022.1063299

**Published:** 2022-11-01

**Authors:** Andrea Romigi, Cinzia Costa, Arjune Sen, Filippo Sean Giorgi

**Affiliations:** ^1^Sleep Medicine Center, IRCCS Neuromed Istituto Neurologico Mediterraneo, Pozzilli, Italy; ^2^Neurology Clinic, Department of Medicine and Surgery, University of Perugia-S. Maria della Misericordia Hospital, Perugia, Italy; ^3^Nuffield Department of Clinical Neurosciences, John Radcliffe Hospital, University of Oxford, Oxford, United Kingdom; ^4^Oxford Epilepsy Research Group, National Institute of Health and Care Research (NIHR) Biomedical Research Centre, Nueld Department of Clinical Neurosciences, John Radcliffe Hospital, Oxford, United Kingdom; ^5^Department of Translational Research and New Technologies in Medicine and Surgery, University of Pisa, Pisa, Italy

**Keywords:** epilepsy, elderly, sleep, dementia, amyloid

Population aging represents a challenge and an opportunity for excellent health care for older people. Epilepsy is the third most common neurological disorder in older adults. In addition, people older than 65 years are the fastest growing demographic worldwide. With the incidence of epilepsy increasing progressively after 50 years and more people with even complex epilepsy thankfully living into adulthood, the prevalence of epilepsy in older age is destined to increase markedly (1). This older cohort has, though, received much less attention than younger people with epilepsy (1).

The causes of epilepsy in older people are mainly ischemic (37%) or hemorrhagic (12%) stroke, tumors (13%) and neurocognitive disorders (12%) (2). With this in mind, DiFrancesco et al. provide a practical and easy-to-use tool to support routine clinical activity for late-onset epilepsy in older people. The authors emphasize the role of the first-level examinations (medical history, blood tests, cardiovascular, and respiratory assessments), neuroimaging (MRI, PET) and electroencephalography (EEG), with thought given to the particular value of 24-h ambulatory EEG (A-EEG) and video-EEG for differential diagnosis of seizures. Nevertheless, this first-level evaluation does not allow identification of the cause of late-onset epilepsy in a high percentage of subjects (about 15%) where neuropsychology, CSF neurodegeneration biomarkers (at least Aß-42 amyloid, tau, and phosphorylated tau) and nuclear medicine (SPECT or PET) may allow distinguishing neurodegenerative and autoimmune etiologies.

To complement the work of DiFrancesco et al., in their retrospective analysis, Morano et al. compare clinical and electrophysiological features of ambulatory 24-h EEG in autoimmune epilepsies (AE) and late-onset epilepsy of unknown origin (LOEU). These authors found that high-frequency focal autonomic seizures should increase the suspicion of an underlying AE, highlighting the role of A-EEG, an easy, inexpensive and non-invasive tool to reduce the diagnostic delay. Potential vascular etiologies were evaluated by Hsieh et al. who detail a large study of the role of middle cerebral artery territory infarction in post-stroke epilepsy. In this work, 774 subjects with MCA infarction, when compared with 1,064 non-MCA patients, showed a higher hazard ratio for post-stroke epilepsy (2.06; 95% CI: 1.33–3.19). Significant factors associated with epilepsy were atrial fibrillation, depression, stroke severity (NIHSS scores of ≥ 16), and alertness on arrival.

Continuing the vascular theme, Tartara et al. thoroughly evaluated the role of leukoaraiosis in people with LOEU. In another large case series, the authors show that LOEU has a better prognosis than symptomatic forms and the presence of leukoaraiosis significantly, and independently, worsens seizure prognosis.

It is well-known that the relationship between sleep and epilepsy is bidirectional and can be challenging (3). The helpful review by Szabo et al. discusses the sleep-related mechanisms underlying epileptogenicity in Alzheimer's Disease (AD) and, conversely, dementia onset in epilepsy to clarify how such events might modulate AD progression. Given that LOEU has been associated with cognitive deterioration and cerebral Aß amyloidopathies (1, 4), molecular, synaptic, and network involvement may be associated with increased epileptogenic activity before neurodegeneration (4–6). The important role of sleep is also highlighted by Csernus et al. These authors review the clinical significance of subclinical epileptiform activity (SEA) and why and how we should diagnose this electrophysiological pattern. SEA is more evident in deep sleep (N3), over the left temporal region and may impair cognitive functions. Overnight EEG recording seems highly sensitive to detecting epileptiform discharges and this could provide fertile ground for future research as well as clinical application. Finally, the authors strongly encourage a consensus panel to obtain unified neurophysiology guidelines to understand better the role of SEA in AD and their potential treatment (7).

Several studies have suggested the epileptogenic potential of Aβ amyloid as a main element driving the cognitive decline and the etiology of late-onset epilepsy in both animal models and clinical studies (4–6, 8–12). del Pozo et al. explore the role of aged rodents with seizures and epilepsy models to potentially identify gaps in LOEU pathophysiology as well as discussing how aging-related neurological disease models may offer more realistic testing for new treatment options.

Finally, in a large retrospective study, Qi et al. evaluate the treatment outcomes of different anti-seizure medications (ASMs) as monotherapy in newly diagnosed epilepsy in older people. They confirm positive initial responses to ASM outcomes and that those with LOEU had higher probabilities of early seizure freedom than those with secondary epilepsy.

As we expected, work in this interesting and important Research Topic raises more questions than answers, We are still far from clarifying the bidirectional relationships between epilepsy/subclinical epileptiform discharges and cognitive decline; the powerful modulating role of sleep; which ASMs should be started in older people with differing etiologies for seizures and if starting ASM after first seizure (13) and certain treatments [e.g., levetiracetam (14)] may also influence the cognitive trajectory. The array of work presented in this Special Edition of Frontiers perhaps serves best to emphasize the need for more large, prospective and translational studies in older people with seizures.

## Author contributions

AR and FG: writing and critical revision. CC and AS: critical revision. All authors contributed to the article and approved the submitted version.

## Conflict of interest

The authors declare that the research was conducted in the absence of any commercial or financial relationships that could be construed as a potential conflict of interest.

## Publisher's note

All claims expressed in this article are solely those of the authors and do not necessarily represent those of their affiliated organizations, or those of the publisher, the editors and the reviewers. Any product that may be evaluated in this article, or claim that may be made by its manufacturer, is not guaranteed or endorsed by the publisher.
